# The Influence of Bereavement on Body Mass Index: Results from a National Swedish Survey

**DOI:** 10.1371/journal.pone.0095201

**Published:** 2014-04-23

**Authors:** Aldair J. Oliveira, Mikael Rostila, Jan Saarela, Claudia S. Lopes

**Affiliations:** 1 Laboratory of Social Dimensions Applied to Physical Activity and Sport (LABSAFE), Department of Physical Education and Sports, Rural Federal University of Rio de Janeiro, Seropédica, Brazil; 2 Centre for Health Equity Studies (CHESS), Stockholm University/Karolinska Institutet, Stockholm, Sweden; 3 University of Helsinki, Helsinki, Finland and Åbo Akademi University, Vasa, Finland; 4 Department of Epidemiology, Institute of Social Medicine, Rio de Janeiro State University, Rio de Janeiro, Brazil; Federal University of Rio de Janeiro, Brazil

## Abstract

**Background:**

Previous findings suggest that the loss of a family member is associated with health and mortality. The purpose of this study was to investigate the association between bereavement experiences and BMI, and whether there are socio-demographic differences in this association.

**Objective:**

To investigate the association between bereavement experiences and BMI, and whether there are socio-demographic differences in this association.

**Methods:**

We used cross-sectional data with retrospective questions from the Swedish Level of Living Survey (LNU) of 2000, including 5,142 individuals. The bereavement experiences examined in the study include the loss of a sibling, a parent or a spouse, and time since the death of a parent. BMI (kg/m^2^) was calculated using self-reported measurements of weight and height. The association between bereavement and BMI was evaluated through linear regressions.

**Results:**

After controlling for possible confounders, most of the models detected an association between bereavement and BMI. The fully-adjusted model showed that loss of parents was associated with a 0.45 increase in BMI (SE = 0.20). The effect also seemed to be dependent on time since the loss and social class position.

**Conclusions:**

The present study is the first to examine associations between different types of familial losses and BMI. We find an association between the death of a family member and BMI, but it appears to be related to time since the death, type of bereavement experience and social class.

## Introduction

The trend of increasing body mass index (BMI) is one of the major public health problems worldwide. BMI has been associated with various socio-demographic, behavioral and psychological factors, such as age, smoking cessation, depression, diet and exercise habits [1]. Additionally, several studies suggest that BMI is influenced by stress and stressful life events [2], [3] as well as poor mental health [4]. Losing a family member represents just such a stressful life event and may adversely impact BMI through psycho-physiological stress mechanisms [5]. To our knowledge, few previous studies have examined whether familial loss is associated with BMI.

Losing a family member is one of life's most difficult experiences. Several studies have suggested that the death of a family member has an adverse effect on physical and mental health [6]–[8]. It has also been revealed that bereavement has implications for mortality [9]–[11]. For instance, Rostila et al. [9] used a longitudinal design based on the total Swedish population register showed that the death of a child was associated with overall increased mortality in parents. They also found that the death of a parent was associated with children's mortality risk [12]. Major explanations for the implications of bereavement for health involve increased chronic and acute stress [13] and poor mental well-being [4].

Some studies have examined the association between bereavement and BMI. One study investigated the influence of childhood parental loss and cardiovascular risk factors, including BMI. The authors reported that this bereavement experience was associated with a decrease in BMI [14]. Another study found that prenatal stress related to maternal bereavement was associated with increased risk of BMI in offspring during school age [15]. Yet studies examining the relationship between BMI and loss of a family member are scarce. Moreover, there are conflicting hypotheses as to whether bereavement contributes to an increase or decrease in BMI [14], [15]. Finally, few studies have examined whether different bereavement experiences (e.g, loss of a spouse, parent and sibling) have varying effects on BMI.

Despite the fact that stress could be one explanation for the implications of bereavement for BMI, we could also postulate different psychosocial pathways. Considering that the death of a family member (such as a parent or sibling) often represents the loss of the longest and most intimate relationship in a person's life, a survivor's psychological well-being could be negatively affected, which, in turn, could result in loss of appetite and a decrease in BMI (acute mechanism) [16]. However, bereavement may also lead to binge eating and poor eating habits that serve to help a survivor dull his/her senses, relieve sorrow and escape emotional pain, which may lead to an increase in BMI [17]. In addition, simultaneous bereavement experiences may represent an additional negative impact on BMI due to cumulative grief, which may lead to higher stress levels [18]. Finally, lack of social support could additionally increase stress following bereavement, which could lead to increases as well as decreases in BMI [19].

There are reasons to expect that the association between a family member's death and the BMI of surviving family members will differ as a function of socio-demographic characteristics. Regarding sex, spousal death might be more detrimental to men's than to women's health, given that women's support networks often include close friends and relatives as confidantes, whereas men typically name their wives as their main source of support [20]. On the other hand, women are more likely than men are to eat in response to negative emotions [21]. Among family members, the influence of bereavement on BMI could also be age-related, as death becomes more natural with increasing age [22]. Thus, the loss of an older relative could lead to less severe consequences for BMI. The BMI of bereaved individuals from the lower social classes could be more affected due to their lower levels of social support, compared to those in the higher social classes [23].

In general, our hypothesis is that bereavement is associated with increases in BMI, although socio-demographic differences may exist. Therefore, the present study has two aims: (1) to examine the association between familial loss (i.e., loss of a sibling, parent or spouse) and BMI, and (2) to investigate whether the association between bereavement and BMI is dependent on socio-demographic characteristics (e.g., sex, age and social class).

## Methods

### Design and study population

Ethical approval for this research was granted by The Swedish Research Council (Vetenskapsrådet, ethical approval number 2012/1260-31). The empirical analyses are based on The Swedish Level of Living Survey (LNU). The survey is conducted through face-to-face interviews with a representative sample of the Swedish population aged 18 to 75 years. The survey has a panel design, but to maintain the survey's representativeness of the Swedish population at any given time, each survey wave also includes younger generations and newly arrived immigrants. The present study uses a cross-sectional design, including 5,142 individuals who participated in the LNU 2000 wave.

### Outcome

#### Body Mass Index

BMI (kg/m2) was calculated based on self-reported measurements of weight and height. It was corrected with the algorithms suggested by Nyholm and colleagues [24], using the Swedish population to correct for self-report bias: in the present case the fact that individuals tend to underestimate their weight and overestimate their height [25]. The following algorithms were adjusted for age and calculated separately by sex:

For men: BMIC  =  −0.202 +1.005 × BMI^SR^ +0.014 × age

For women: BMIC  =  −0.713 +1.023 × BMI^SR^ +0.019 × age

BMI is the value of the self-reported BMI, and age was in years. BMIC is the new value of BMI.

### Exposure

#### Bereavement

Three types of bereavement were evaluated: the loss of a sibling, a parent or a spouse. The loss of a *sibling* was examined using a dichotomous variable (yes or no). The loss of *parents* was evaluated using the following variable: non-bereaved, loss of father, loss of mother and loss of both. Additionally, time since the loss of parents was also evaluated. Time since the loss of a mother or a father was investigated as follows: “never”, “three years ago or more recently”, “between 4 and 8 years ago”, “more than 8 years ago”. Finally, the loss of a *spouse* was evaluated using a dichotomous variable (yes or no).

### Covariates

The covariates included in the analysis were sex, age, marital status, social class and emotional support. Marital status was measured using two categories: “married/cohabiting” and “non-married”. We measured social class based on the Swedish socioeconomic classification (SEI), which included the following categories: “non-manual”, “manual”, and “self-employed” (Note: students and unemployed were not included in the SEI). Measurement of emotional support was based on the question: “*Sometimes we need other people's help and support. Do you have a family member or friend who helps out if you need to talk to someone about personal problems*?” (Note: also see Medical Outcomes Study Social Support Survey) [26]. The respondents provided a dichotomous answer to these questions by answering “yes” or “no”.

### Modeling strategy

To examine whether different sources of bereavement (e.g., loss of sibling, loss of parents and loss of spouse) are associated with BMI, we analyzed data from LNU 2000. In the first approach, exposure information was included as independent variables and BMI as the dependent variable (Model 1- the unadjusted model). Subsequently, other models were adjusted to represent confounding scenarios in four different adjusted models that included the covariates. Model 2 was adjusted by sex, age, and social class. Models 3, 4 and 5 added other variables to the previous model in a stepwise manner: Model 3 added marital status as a variable to the basic confounders in Model 2; Model 4 added number of siblings to Model 3; and Model 5 (the fully adjusted model) added emotional support to Model 4. In a second approach, to evaluate whether the impact of bereavement on BMI differs among socio-demographic variables, we also tested the interactions between the bereavement variables and the social demographic variables (e.g., sex, age and social class) in relation to BMI. All of the models were fitted separately by bereavement variable, and the non-bereaved were the reference group in the analysis.

### Statistical analysis

We calculated descriptive summary statistics by calculating the means and standard deviations of the continuous variable and the frequencies for count variables according to the socio-demographic variables. To estimate the coefficients (β) and their estimation error (SE), we ran linear regression models. The model parameters were estimated using the R workspace version 2.15.0.

## Results

Among women, the mean age was 44 years (standard deviation of 15.5) and the BMI mean was 23.7 kg/m^2^ (standard deviation of 4.1). Among men, the mean age was 42 years (standard deviation of 15.5) and the BMI mean was 25.0 kg/m^2^ (standard deviation of 3.4). A summary of the descriptive statistics for the bereavement variables according to sex and social class is shown in Table 1.

**Table 1:** Descriptive statistics of the bereavement variables according to sex, age and social class in LNU.

Bereavement variables	Number of observations - n(%)									
	Total	Sex		Age (years)			Social class			
		Men	Women	18 to 38	39 to 59	60 to 75	Manual	Non-manual		Self-employed
**Loss of sibling**										
No	4379 (98.6)	2142 (86.2)	2237 (87.9)	1845(96.5)	1587(87.6)	510 (58.3)	1745(86.0)	2176 (88.6)		379 (87.6)
Yes	655 (1.4)	345 (13.8)	310 (12.1)	66 (3.5)	224(12.4)	365 (41.7)	284 (14.0)	225 (9.4)		54 (12.4)
**Time of losing Mother**										
No	3375 (65.8)	1632 (64.1)	1743 (67.6)	1945(95.0)	1323(64.2)	1007(52.5)	1373(65.3)	1653(66.8)		279(61.2)
0–3 years	214 (4.2)	113 (4.4)	101 (3.9)	18 (0.9)	134 (6.5)	62 (3.2)	87 (4.1)	107 (4.3)		18 (3.9)
4–8 years	253 (4.9)	144 (5.7)	109 (4.2)	18 (0.9)	133 (6.4)	102 (5.3)	98 (4.7)	126 (5.1)		31 (6.8)
8 years or more	1284 (25.0)	658 (25.8)	626 (24.3)	65 (3.2)	472 (22.9)	747 (39.0)	544 (25.9)	590 (23.8)		128 (28.1)
**Time of losing Father**										
No	2628 (63.6)	1274(61.9)	1354 (65.2)	1801(98.2)	800 (57.7)	27 (4.9)	1079(63.1)	1301(65.2)		198(55.6)
0–3 years	155 (3.7)	75(3.6)	80 (3.8)	5 (0.3)	93 (6.7)	57 (10.3)	67 (3.9)	76 (3.8)		11 (3.1)
4–8 years	214 (65.2)	127(6.3)	87 (4.2)	8 (0.4)	108 (7.8)	98 (17.7)	81 (4.7)	104 (5.3)		27 (7.6)
8 years or more	1138 (27.5)	581(28.2)	557 (26.8)	21 (1.1)	386 (27.8)	371 (67.1)	486 (28.3)	513 (25.7)		120 (33.7)
**Loss of spouse**										
No	4968 (96.6)	2406 (95.8)	2562 (98.4)	2047(99.9)	2031(98.1)	890 (87.9)	2029(96.3)	2408(97.1)		442(96.9)
Yes	174 (3.4)	134 (4.2)	40 (1.6)	2 (0.1)	39 (1.9)	133 (12.1)	78 (3.6)	73 (2.9)		14 (3.1)
**Loss of parents**										
No	2393 (47.0)	1161 (45.5)	1232 (48.6)	1733(85.4)	652 (31.8)	8 (0.8)	991(47.7)	1176(47.8)		180(39.8)
Lose father	956 (18.8)	489 (19.2)	467 (18.4)	197 (9.7)	661 (32.3)	98 (9.7)	369(17.6)	468(19.0)		96(21.2)
Lose mother	233 (4.6)	120 (4.7)	113 (4.5)	66 (3.2)	148 (7.2)	19 (1.9)	87(4.3)	124(5.0)		18(4.0)
Lose both	1504 (29.6)	782 (30.6)	722 (28.5)	34 (1.7)	586 (28.7)	884 (87.6)	631(30.4)	693(28.2)		158(35.0)

LNU =  The Swedish Level of Living Survey.

Overall, the models showed associations between familial loss and BMI. Furthermore, these models also showed a decrease in the coefficient values across the bereavement variables after adjustment for socio-demographic variables. However, adding the other covariates did not significantly change the model coefficients.

Table 2 showed a significant (p<0.05) association between those who were exposed to parental death and BMI. In this sense, the fully-adjusted models (Model 5) showed that the loss of both parents increased the BMI of bereaved people by 0.45 (SE = 0.20) BMI units, on average. The other types of bereavement did not show significant associations when models were adjusted for confounders, although there was a tendency toward significance among individuals who had lost a spouse.

**Table 2:** Regression coefficients and standard errors estimated from multiple linear regressions for the association between bereavement and BMI.

Bereavement	Model 1 β (SE)	Model 2 β (SE)	Model 3 β (SE)	Model 4 β (SE)	Model 5 β (SE)
**Loss of sibling**					
Yes	1.00 (0.16)^a^	0.22 (0.17)	0.21 (0.17)	0.19 (0.18)	0.20 (0.18)
**Loss of spouse**					
Yes	0.85 (0.30)^a^	0.10 (0.30)	0.62 (0.33)^c^	0.69 (0.36)^c^	0.70 (0.36)^c^
**Loss of parents**					
Lose father	0.93 (0.14)^a^	0.05 (0.15)	0.04 (0.16)	0.01 (0.16)	0.01 (0.17)
Lose mother	1.08 (0.26)^a^	0.34 (0.26)	0.30 (0.26)	0.41 (0.27)	0.41 (0.27)
Lose Both	1.70 (0.12)^a^	0.50 (0.19)^b^	0.47 (0.19)^b^	0.44 (0.20)^b^	0.45 (0.20)^b^

The outcome was the BMI (kg/m^2^) in LNU 2000. The covariates were included based on informations provided by LNU 2000. The Swedish Level of Living Survey. The models were fitted separately by bereavement variable (reference group in bereavement variables: individuals who have not had such bereavement experience).

a p<0.001; ^b^p<0.05; ^c^p<0.10 n =  number of observations; β =  coefficient estimated; SE =  estimation error.

Model 1: Bereavement variable.

Model 2: Bereavement variable + sex + age + social class.

Model 3: Bereavement variable + marital status + age + social class + marital status.

Model 4: Bereavement variable + marital status + age + social class + marital status + number of siblings.

Model 5: Bereavement variable + age + social class + marital status + number of siblings + emotional support.

Regarding time since the loss of parents in relation to BMI (Table 3), the models showed significant associations (p<0.05), primarily 4–8 years since the loss. Having lost a mother or a father 4–8 years ago was associated with a higher average BMI value (0.91 kg/m^2^ for the mother; SE = 0.27, and 0.82 kg/m^2^ for the father; SE = 0.32).

**Table 3:** Regression coefficients and standard errors estimated from multiple linear regressions for the association between bereavement time or frequency and BMI.

Bereavement	Model 1 β (SE)	Model 2 β (SE)	Model 3 β (SE)	Model 4 β (SE)	Model 5 β (SE)
**Time of losing mother**					
0–3 years	0.78 (0.27)^b^	−0.02(0.27)	−0.04 (0.27)	0.02 (0.28)	0.02 (0.30)
4–8 years	1.61 (0.24)^b^	0.79 (0.25)^b^	0.78 (0.24)^b^	0.90 (0.27)^b^	0.91 (0.27)^a^
8 years or more	1.38 (0.14)^b^	0.43 (0.16)	0.41 (0.18)	0.40 (0.18)^b^	0.40 (0.18)^b^
**Time of losing father**					
0–3 years	0.89 (0.30)^c^	−0.27(0.33)^c^	−0.29 (0.33)^c^	−0.26 (0.36)^c^	−0.25 (0.35)^b^
4–8 years	1.86 (0.24)^a^	0.70 (0.28)^b^	0.71 (0.30)^b^	0.79 (0.32)^b^	0.82 (0.32)^b^
8 years or more	1.66 (0.15)	0.37 (0.23)	0.35 (0.23)	0.30 (0.23)	0.31 (0.23)

The outcome was the BMI (kg/m^2^) in LNU 2000. The covariates were included based on informations provided by LNU 2000. The Swedish Level of Living Survey. The models were fitted separately by bereavement variable (reference group in bereavement variables: individuals who have not had such bereavement experiences).

a p<0.001; ^b^p<0.05; ^c^p<0.10 n =  number of observations; β =  coefficient estimated; SE =  estimation error.

Model 1: Bereavement variable.

Model 2: Bereavement variable + sex + age + social class.

Model 3: Bereavement variable + marital status + age + social class + marital status.

Model 4: Bereavement variable + marital status + age + social class + marital status + number of siblings.

Model 5: Bereavement variable + age + social class + marital status + number of siblings + emotional support.

Table 4 showed the interactions between bereavement experiences and socio-demographic variables (e.g., sex, age and social class). Especially among age and social class, most of the results were significant (p<0.05). Significant (p<0.05) interactions were found between bereavement experiences and age, except among those who lost a spouse. However, the magnitudes of these interaction effects were small (varied between: β =  −0.03; SE = 0.01 and β = −0.04; SE = 0.01). The interaction between loss of a family member and social class showed that the loss of a father among non-manuals was inversely associated with BMI, compared with those who had the same bereavement experience but were in the manual class (−1.43 kg/m^2^on average; SE = 0.24). Table 4 showed similar results among other kinds of parental loss (i.e., loss of mother and loss of mother and father). We did not find significant (p<0.05) interactions between familial loss and sex.

**Table 4:** Regression coefficients and standard errors estimated from interactions between bereavement and socio-demographic variables on BMI.

	Coefficient Estimated - β (SE)			
Bereavement	socio-demographic variables			
	**Sex**	**Age**	**Social class**	
			Manual	Self-employed
**Loss of sibling**	−0.24 (0.29)	−0.03 (0.01)^b^	−0.54 (0.31)	−0.36 (0.50)
**Loss of spouse**	0.63 (0.74)	−0.03 (0.03)	0.19 (0.63)	−0.85 (1.18)
**Loss of parents**	–	–	–	–
Loss of father	0.09 (0.22)	−0.04 (0.01)^a^	−1.43 (0.24)^a^	−0.46 (0.43)
Loss of mother	0.32 (0.37)	−0.03 (0.01)^a^	−0.96 (0.39)^b^	0.07 (0.92)
Loss of both	0.31 (0.20)	−0.04 (0.01)^a^	−0.58 (0.22)^b^	−0.46 (0.34)

The outcome was the BMI (kg/m^2^) in LNU 2000 - The Swedish Level of Living Survey.*The reference categories for the analysis are women for gender, non-manual for social class and no bereaved for all bereavement variables. The models were fitted separately by bereavement variables and social demographic ones. The models were adjusted by marital status, number of sibling and emotional support. ^a^p<0.001; ^b^p<0.05; β =  coefficient estimated SE =  estimation error.

## Discussion

The present study examined three different types of familial loss (e.g., loss of sibling, parents and spouse) and their association with BMI. Moreover, we studied whether the association between bereavement and BMI might differ as a function of sex, age and social class. Our results largely confirmed that bereavement might increase BMI, although some of our findings also suggested that familial loss is associated with a BMI decrease among those who lost their parents more than seven years ago. Furthermore, we found interesting interactions between familial loss and socio-demographic variables in relation to BMI, thus highlighting that socio-demographics may play a role in the association between bereavement and BMI.

Our findings highlighting an association between bereavement and BMI may reflect overeating among bereaved people. Overeating could be explained by psychobiological mechanisms [27]. Bereaved people may use food as “self-medication” because sweet tastes have mood-elevating and pain-suppressing properties [28]. Thus, food-related sensory signals, including sight, smell, taste, and texture, and cognitive-affective processes may influence the eating behavior of bereaved individuals through psychobiological mechanisms [27].

Looking at the results on loss of a sibling, the association between a sibling's death and BMI was not significant. However, previous literature on bereavement has reported that the death of a sibling may have a significant impact on the individual when it involves the loss of a companion and a source of emotional and practical support [10], [29].

Regarding parental loss, our results showed a significant, although rather weak, association between loss of a mother and loss of both parents, respectively, and BMI increase. Additionally, our models also revealed a time-dependent relationship, showing that a mother's death four years ago or more was associated with an increase in BMI, while a father's death between four and eight years ago was related to an increase in BMI. However, we also found a BMI decrease among those who lost their father three years ago or more recently. These results suggest that longer-term mechanisms may be involved in explaining the association between parental loss and BMI. In relation to acute mechanisms, elevated cortisol levels have been associated with stressful life events [30], which, in turn, could be related to patterns of decreased caloric intake after parental death that affect body weight [31], [32]. Another acute bereavement response is loss of appetite during the first months, which could be reflected in a decrease in BMI [16]. Regarding distal mechanisms, loss of a parent entails loss of a caregiver and a unique attachment figure who typically provides many kinds of social support. Although other social connections, such as intimate partners, act in the social support system as well, it is possible that some kinds of support cannot be completely covered by other social network members over time. In this sense, the persistence of lower social support over a longer time period could be related to chronic stress, and consequently, contribute to weight gain [33]. In addition, the loss of a parent also involves the loss of social control over health behaviors, such as physical inactivity and poor eating habits [34].

We did not find associations between loss of a spouse and BMI, although the results suggested a tendency toward significantly higher BMI among individuals who had lost a spouse. Three previous studies have shown an inverse association between marital loss and BMI. A study conducted with men in the US showed that marital disruption led to a BMI decrease in male health professionals [35]. Another study conducted with women in the US showed a decrease in BMI associated with divorce or widowhood [36]. Nevertheless, Oliveira et al. [37], using a Swedish population, found an inverse association between divorce or widowhood and BMI among women only. Although there is some evidence for a relationship between marital disruption and BMI, it is mediated by eating habits [35], [36] in both sexes, and it is plausible that men who experience spousal death decrease their intake of vegetables and other foods requiring preparation skills, and instead consume more convenience foods. However, it is also conceivable that mealtimes serve as a reminder, especially of recent loss. Dining is a common bond, and after having shared meals with another person for many years, it may be painful to dine alone or without the loved one [16]. Although previous studies have found associations between loss of a spouse and BMI we cannot make any definite conclusions about a relationship based on the non-significant results found in this study. Further researches using other sources of data are needed to confirm whether there is an association between the loss of a spouse and BMI in Sweden.

Furthermore, we did not find significant interactions between bereavement and sex and BMI. This indicates that the relations between these bereavement experiences and BMI were the same across both sexes. Nevertheless, postulating sex differences is plausible. In this sense, we suggest that these results could reflect siblings' smaller social network as compared with women's [20].

Our results must be interpreted in relation to some potential methodological limitations. The first limitation involves the relatively low number of individuals who had bereavement experience. Consequently, some models returned large estimation errors reflecting problems with statistical power. Nevertheless, in the evaluation of model adjustments based on regular diagnostic plots, we found that our statistical models were appropriately fitted. Second, because the study design was based on access to BMI data at only one point in time, it was not possible to evaluate possible changes in BMI related to bereavement experience. Third, BMI was based on self-reports of weight and height rather than externally administrated measurements, which are more reliable. It is possible that individuals underestimated their weight and overestimated their height. Our use of an algorithm, however, may have corrected for this information bias and minimized this problem. Fourth, there was a possibility of selective responses in the survey, because people with recent familial loss may have been more reluctant to participate in the survey. Finally, we had no information on stress and limited information on eating behavior. Consequently, we could not test whether a possible link between these two factors may have explained some of the patterns in our findings.

To our knowledge, our study was the first to show associations between different types of familial loss and BMI. Future studies should also examine in more detail the mechanisms linking bereavement and BMI and whether these mechanisms differ by subjects' socio-demographic background.
